# Ruthenium on Carbonaceous Materials for the Selective Hydrogenation of HMF

**DOI:** 10.3390/molecules23082007

**Published:** 2018-08-11

**Authors:** Stefano Cattaneo, Hadi Naslhajian, Ferenc Somodi, Claudio Evangelisti, Alberto Villa, Laura Prati

**Affiliations:** 1 Dipartimento di Chimica, Università degli Studi, di Milano, via Golgi 19, I-20133 Milano, Italy; stefano.cattaneo2@unimi.it (S.C.); alberto.villa@unimi.it (A.V.); 2 School of Chemistry, Collage of Science, University of Tehran, P.O. Box 14155-6619, 1417466191 Tehran, Iran; hnhajian88@ut.ac.ir; 3 Centre for Energy Research, Hungarian Academy of Science, Department of Surface Chemistry and Catalysis, Konkoly-Thege M. street 29-33, 1121 Budapest, Hungary; somodi.ferenc@energia.mta.hu; 4 National Council of the Research, CNR-ISTM, Via G. Fantoli 16/15, 20138 Milan, Italy; claudio.evangelisti@istm.cnr.it

**Keywords:** Ruthenium, carbon, nanofiber, HMF, DMF, hydrogenation

## Abstract

We report the use of Ru catalysts supported in the activated carbon (AC) and carbon nanofibers (CNFs) for the selective production of liquid fuel dimethylfuran (DMF) and fuel additives alkoxymethyl furfurals (AMF). Parameters such as the reaction temperature and hydrogen pressure were firstly investigated in order to optimise the synthesis of the desired products. Simply by using a different support, the selectivity of the reaction drastically changed. DMF was produced with AC as support, while a high amount of AMF was produced when CNFs were employed. Moreover, the reusability of the catalysts was tested and deactivation phenomena were identified and properly addressed. Further studies need to be performed in order to optimise the stability of the catalysts.

## 1. Introduction

Over the last few years, the world has been passing through a crucial moment concerning the dependence on oil as its main source of chemicals and energy. The diminishing of the reserves combined with the increase of the world’s demand has led to the necessity to find new sustainable resources in order to gradually replace oil in both the chemical and energy fields [1]. Biomass nowadays represents the most attractive carbon feedstock, since it is abundant, widespread and inexpensive [2,3,4,5,6,7]. Biomass is biological material derived from living or recently living organisms. In the chemical and energy context, this word is often used to indicate non-edible plant-based materials such as grass, wood, straw, crop residue, agricultural, and food waste. The main constituents of plant biomass (lignocellulosic biomass) used as a source of chemicals and fuels are cellulose, hemicelluloses, and lignin, and they represent 75% of the 170 billion metric tons of the total biomass production per year by photosynthesis [8].

One of the most important products directly derived from lignocellulosic biomass is 5-hydroxymethylfurfural (HMF). HMF can be found in nature in different plants and foods [9] and it is also formed during the thermal decomposition of carbohydrates, especially as an intermediate in the caramelization of sugars [10]. The attractiveness of this “sleeping giant”, as it has been called in the last few years, is due to the simultaneous presence of a carbonyl and alcoholic group and an aromatic furan ring. Moreover, HMF can not only be produced with a high selectivity by the dehydration or isomerization of fructose, but, recently, new routes have been reported using cellulose directly as a starting material [9,11,12]. Currently, a wide range of HMF derivatives is reported to be potentially suitable for use in several sectors of chemical industries and as biofuels. Among all these, the HMF hydrogenation products are of particular interest. 

**Figure 1:**
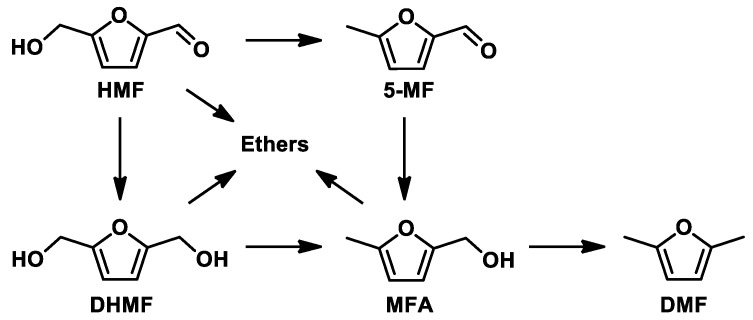
The reaction mechanism for the conversion of HMF using alcohols as a solvent.

The hydrogenation reaction of HMF involves numerous intermediate products and the mechanism for their formation is rather complex. The most common products are reported in Figure 1. The dominant pathways are the hydrogenation of the carbonyl group and the hydrogenolysis of the C-O bond, but other secondary reactions are widely reported in the literature, such as decarboxylation [13], ring hydrogenation [14,15], ring opening [16,17], and etherification when using alcohol as a solvent [18].

Among all the HMF hydrogenation products, 2,5-dimethylfuran (DMF) and HMF-derived ethers (alkoxymethyl furfurals, AMF) have recently attracted a high amount of attention in the scientific community. DMF is a well-known potential biofuel that possesses extremely interesting properties, such as a high research octane number (RON), a low water solubility, and a high energy density [19]. Various studies have been performed in order to increase the yield of this useful product. An initial yield of 79% was obtained by Dumesic et al. in a flow reactor loaded with CuRu/C as a catalyst and 1-butanol as a solvent [20]. In the following years, selectivities higher than 95% were obtained by many groups by use of single metal supported catalysts, such as Ru [21,22,23,24,25], Pd [26], Pt [27], Ni [28], and Cu [29] or metal alloys such as AuPd [30], PtCo [31,32], and NiCu [33].

The formation of ethers from HMF and alcoholic solvents (AMF, alkoxymethyl furfurals) is widely reported in the literature, especially as byproducts in the dehydration process of fructose (or hexose in general) when an alcoholic solvent and an acidic solid catalyst are used [34]. Several patents were released in favour of Avantium, who claimed the use of various AMF as potential fuel or fuel additives [35,36]. Recently, various studies have been published concerning the production of AMF, especially with small molecular weight alcohols (since the reactivity decrease increasing the length of the hydrocarbon chain), obtaining high yields by using various types of solid acid catalysts [18,24,37,38,39,40,41].

In this paper, HMF hydrogenation was carried out with a ruthenium catalyst supported by activated carbon (Ru/AC) and two different types of carbon nanofibers (Ru/CNFs-HHT and CNFs-PS) to explore the influence of the support in the reaction activity and selectivity. An alcoholic solvent was used in order to perform a comparative study of the product distribution between DMF and AMF; in particular, 2-butanol was used as it is one of the most used extraction solvents in the fructose dehydration of the HMF process [9,11,20,42], and ether formation has already successfully been reported during HMF and furfural hydrogenation reactions [24,35,43]. The reaction temperature and hydrogen pressure effects were initially studied. Finally, all the catalysts were tested in consecutive reactions to evaluate the eventual deactivation processes and a broad range of characterization techniques such as TPR, TEM, and XPS have been employed to better understand the properties of the catalyst.

## 2. Results and Discussion

### 2.1. Catalysts Characterisation

**Figure 2:**
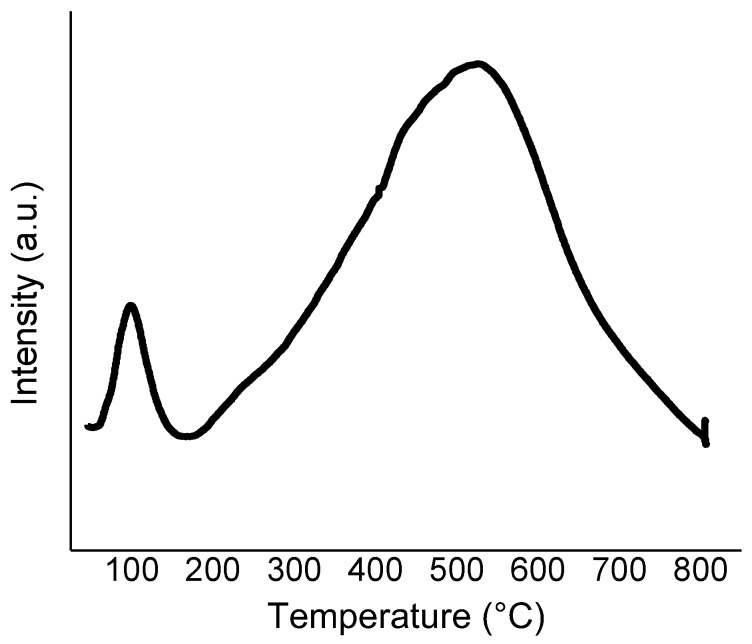
The TPR analysis of the 1 wt% Ru/AC catalyst.

Ru catalysts were prepared by incipient wetness impregnation using activated carbon (AC) and two different carbon nanofibers (CNFs) with a different graphitization grade. A first single temperature programmed reduction analysis (TPR) was performed on the Ru/AC catalyst in order to choose an appropriate reduction temperature for the catalyst activation step (Figure 2). The peak with a maximum at 99 °C corresponds to the reduction of Ru oxide [44,45] while the large one centred at ca. 500 °C can be addressed to the decomposition phenomena of the catalyst with the formation of hydrocarbons from the reduction of the carbon support catalysed by the Ru species [44,46]. Therefore, all the catalysts were activated at a temperature high enough to reduce Ru^n+^ to metallic Ru (200 °C) but not high enough to promote the decomposition phenomena.

**Figure 3:**
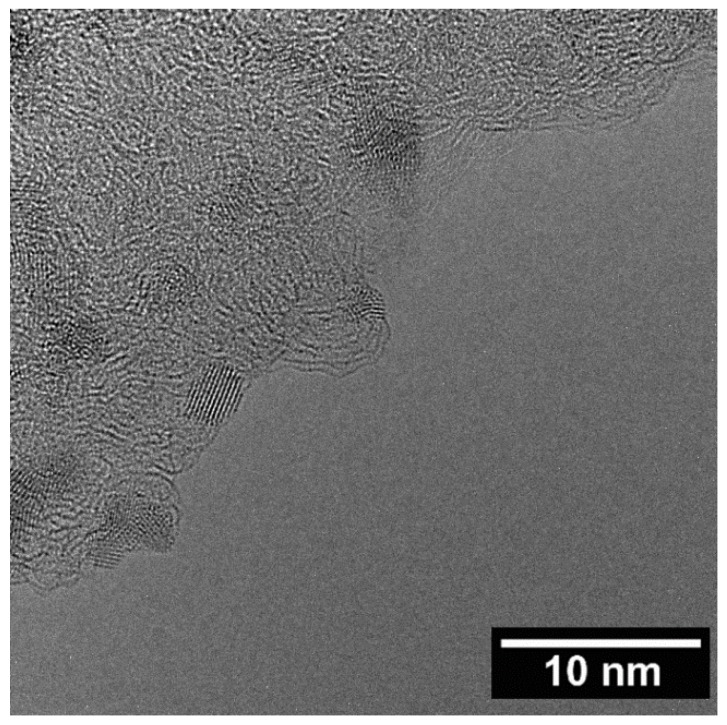
The TEM micrograph of the Ru/AC catalyst.

A transmission electron microscopy analysis (TEM) was performed on the three Ru catalysts supported on AC, CNFs-HHT, and CNFs-PS (Figure 3 and Supplementary Information). A similar average particle size was observed across the catalysts (1.0–1.9 nm), as reported in Table 1. Particle agglomeration was observed on the Ru/CNFs-PS catalyst, and for this reason, the size distribution was much larger compared to the other catalysts.

An X-ray Photoelectron Spectroscopy analysis (XPS) was performed in order to obtain information about the relative amount and oxidation state of the active metal and the presence and abundance of oxygen functionalities on the support surface (Supplementary Information). The abundance of Ru was calculated compared to the carbon (Table 2). Both Ru/CNFs-HHT and Ru/CNFs-PS showed a much lower content of the active metal compared to Ru/AC, most likely due to the presence of Ru particles inside the CNFs channels, considering the similar particle size. Indeed, the EDX analysis on the Ru/CNFs-HHT confirmed, an average, a metal loading of 1.0 ± 0.2 wt% in perfect accordance with the nominal value. Moreover, despite the reduction step, XPS showed that in all the samples, the Ru was present as an Ru^4+^ species (BE of ca. 463 eV), most likely RuO_2_, due to the passivation phenomena that occurs when the catalyst gets in contact with air. The XPS in-situ activation was not possible to perform, thus, it is likely that the passivation occurred simply during the transfer of the catalyst into the XPS spectrometer. In the Ru/AC catalyst, a second peak at ca. 465 eV was observed and it can be assigned to either water molecules bound to the surface of Ru^4+^ species or to the higher oxidation state Ru species [47].

The abundance of oxygen functionalities and the relative contribution of different surface functional groups present on the support was based on the C 1s spectra (Table 2). As expected, the untreated CNFs-PS showed a higher O/C atomic ratio (0.26) compared to the high temperature treated CNFs-HHT (0.18), while the activated carbon was the support containing the least amount of oxygen, with an O/C atomic ratio of 0.15. In all the supports, the predominant oxygen functionalities were C-O species (ca. 44–58%), with only a small amount of carboxylic functionalities (<17%). Two additional peaks were observed in the C 1s spectra at ca. 285 eV and ca. 291 eV, and were assigned to the C-C bonds and to the π-π* transitions in aromatic rings respectively [48].

### 2.2. Temperature and Pressure Effect

**Figure 4:**
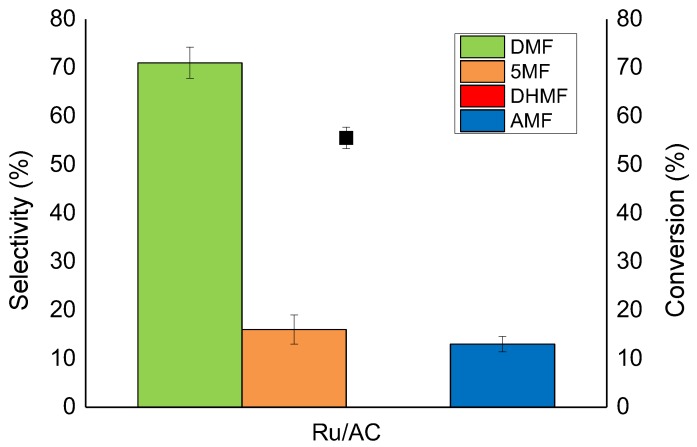
The HMF hydrogenation reaction on Ru/AC catalyst. Reaction conditions: HMF, 0.08 M; substrate/metal = 100 mol/mol; total volume, 15 mL; 150 °C; 20 bar H_2_.

Preliminary studies have been conducted in order to optimize the reaction parameters; for this purpose, a 1 wt% Ru/AC catalyst was used. The HMF hydrogenation reaction is a series of consecutive and parallel reactions (Figure 1) that can take place in a very short time. The first reaction was conducted in 2-butanol as a solvent at 150 °C and 20 bar of H_2_ (Figure 4). After only 1 h, the conversion was 55%, with the main product being DMF (71% of selectivity). The product of the hydrogenolysis of the alcoholic group, 5-MF, was also present in a considerable amount (16% of selectivity). The absence of DHMF and MFA from the reaction mixture indicates that in the present reaction conditions, the hydrogenolysis reaction occurs much faster than the hydrogenation reaction on the Ru/AC catalyst tested. The remaining 13% of the products are a mix of ethers (AMF) derived from the reaction between DHMF and MFA with the solvent (Supplementary Information), while no products of ring hydrogenation/opening were detected. In order to study the product distribution in the absence of the etherification process, the same reaction was performed using tetrahydrofuran (THF) as a solvent. The results (Figure S5) show a similar DMF production after 30% of conversion (ca. 70%). Interestingly, DHMF was the only intermediate product observed. Clearly, the use of an alcoholic solvent strongly affects the product distribution. In particular, in the presence of 2-butanol, DHMF readily converts to AMF in the presence of Ru/AC as a catalyst; on the other hand, when the reaction is performed in the absence of alcohol, the DHMF is not immediately transformed and can be observed in the reaction solution.

**Figure 5:**
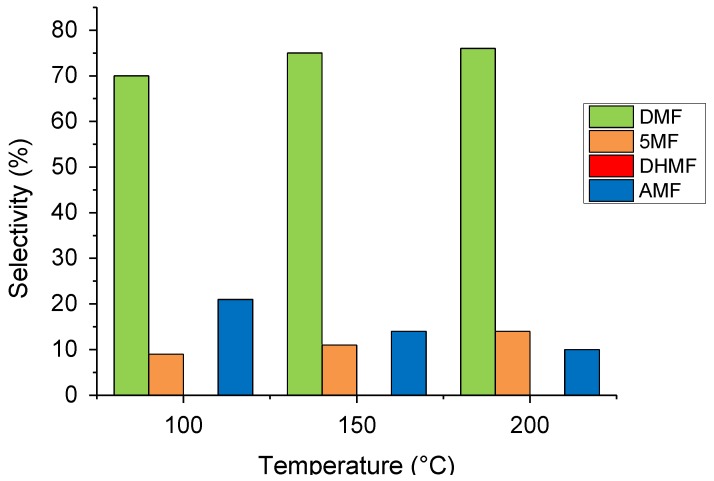
The temperature effect on the HMF hydrogenation reaction. Reaction conditions: HMF, 0.08 M; substrate/metal = 100 mol/mol; total volume, 15 mL; 20 bar H_2_. Selectivity at 60% conversion.

The reaction temperature was varied in a range of 100–200 °C and the different reactions were compared at 60% iso-conversion (Figure 5). On increasing the reaction temperature, as expected, the HMF conversion increased linearly (from 8% to 100% at 100 and 200 °C, respectively, Figure S6). The selectivity, on the other hand, did not change drastically. The DMF and 5-MF production slightly increased at the expense of the products of etherification, showing that the etherification reaction is slightly unfavored at higher temperatures compared to the hydrogenolysis mechanism.

**Figure 6:**
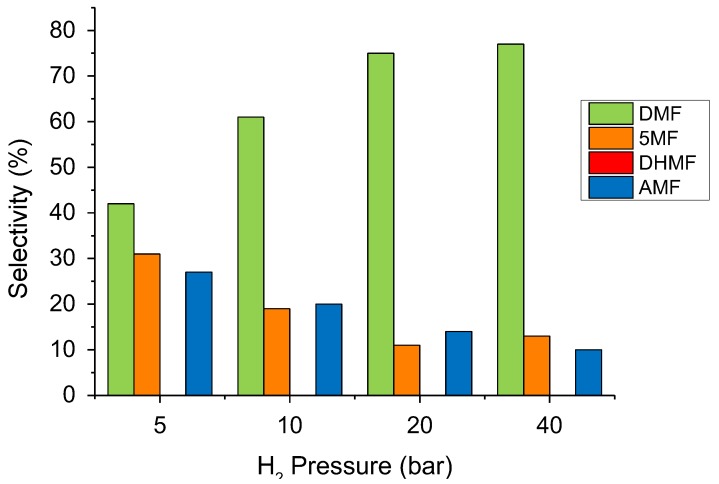
The H_2_ pressure effect on the HMF hydrogenation reaction. Reaction conditions: HMF, 0.08 M; substrate/metal = 100 mol/mol; total volume, 15 mL; 150 °C. Selectivity at 60% conversion.

The hydrogen pressure effect was then investigated from 5 to 40 bar (Figure 6). The HMF conversion increased linearly with the hydrogen partial pressure up to 20 bar (from 14 to 59%, Figure S7), after which a further addition of hydrogen did not significantly change the rate of HMF consumption. At pressures higher than 20 bar, the HMF hydrogenation reaction was, therefore, independent of the concentration of hydrogen. Contrary to temperature variations, the hydrogen pressure has a strong influence on the selectivity of the iso-conversion (Figure 6). The production of DMF increased from 42% to 77%, varying the total pressure from 5 to 40 bar, while both 5-MF and AMF decreased in the same range of the pressure studied. It is clear from these results that the hydrogenation/hydrogenolysis mechanisms are favoured at higher hydrogen pressures, while the etherification reaction prevails at lower pressures.

### 2.3. Support Effect

Carbon nanofibers (CNFs) are widely used as supports for metal nanoparticles due to their high surface area and unique surface chemistry properties. Although they have shown promising results in many hydrogenation reactions, only a few reports have focussed their attention on the selective hydrogenation of HMF [49]. Two commercial CNFs with different oxygen contents have been used to support Ru nanoparticles, specifically CNFs-PS and CNFs-HHT. The former are pyrolytically stripped fibres composed of a thin chemical vapour deposited (CVD) layer of amorphous carbon over a graphitic fishbone core, while the latter are high-temperature treated fibres (3000 °C) where the surface CVD carbon layer is fully graphitised due to the high temperature of the treatment involved.

**Figure 7:**
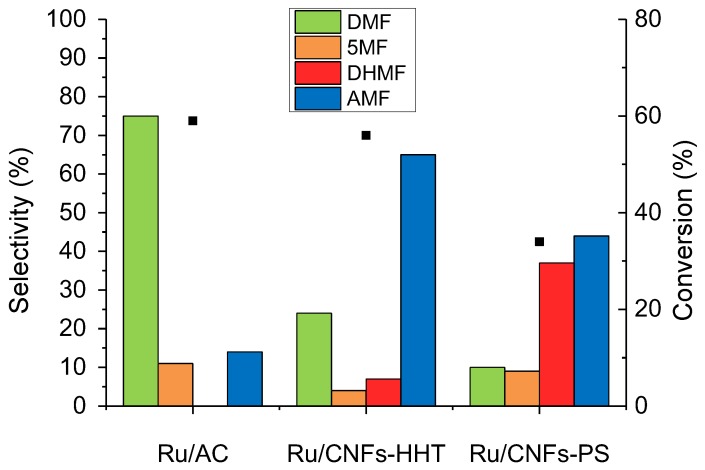
The support effect on the HMF hydrogenation reaction. Reaction conditions: HMF, 0.08 M; substrate/metal = 100 mol/mol; total volume, 15 mL; 150 °C; 20 bar H_2_.

Figure 7 shows the comparison, in terms of activity and selectivity, between the three different catalysts, namely Ru/AC, Ru/CNFs-HHT, and Ru/CNFs-PS. The activity of the Ru supported on AC and CNFs-HHT was very similar (59% and 56% of conversion after 1 h for Ru/AC and Ru/CNFs-HHT respectively) and almost twice as high compared to the Ru/CNFs-PS catalyst (34% conversion). The difference in activity can be ascribed to the presence of particle agglomerations in the Ru/CNFs-PS catalyst as even the particle size is similar (Table 1); these features contributed a large amount to the total amount of Ru in the catalyst but only contributed a small amount to the overall exposed metal surface.

Interestingly, the selectivity of the various products varied drastically simply by changing the nature of the support. DHMF, for example, was produced in a considerable amount (37% selectivity) with the Ru/CNFs-PS catalyst. On the other hand, a very high amount of AMF was produced with the high-temperature treatment of CNFs-HHT (65%). This is unexpected since the production of AMF is usually reported to be correlated to support acidity. Indeed AMFs are obtained in high yield with solid acid catalysts such as Amberlyst-15 [38]. XPS analyses of Ru/AC and Ru/CNFs-HHT (Table 2) revealed similar oxygen contents and the similar distribution of support surface groups. The only relevant difference between the two catalysts was the Ru surface exposure (Ru/C 0.009 for Ru/AC and 0.004 for Ru/CNFs-HHT). Moreover, Ru/CNFs-PS, despite a lower conversion, having a similar Ru surface exposure (Ru/C 0.003), showed a comparable selectivity with Ru/CNFs-HHT. Therefore, a possible explanation of the high selectivity to AMF showed by Ru on CNFs lies on the location of the Ru particles.

### 2.4. Catalyst Reusability

**Figure 8:**
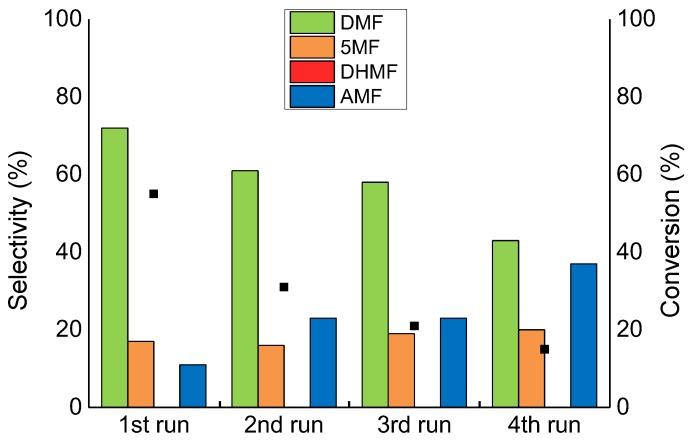
The reusability test on the Ru/AC catalyst. Reaction conditions: HMF, 0.08 M; substrate/metal = 100 mol/mol; total volume, 15 mL; 150 °C, 20 bar H_2_, reaction time, 1 h.

All the catalysts were tested in consecutive reactions to gather information on the catalytic reusability of the synthesised materials. After the first run, the catalyst was filtered from the reaction mixture and used for a second run. Figure 8 shows the reusability test performed on the Ru/AC catalyst. The reaction conversion dropped from 55% to 31% after only 2 uses and it only reaches 15% at the end of the fourth run, clearly showing the presence of deactivation phenomena. The selectivity to DMF decreased linearly after four runs from 72% to 43%, with an increase in selectivity towards AMF from 11% to 37%. The XPS analysis confirmed the presence of adsorbed oxygenated species on the catalyst surface after the reaction (Table 2); the O/C ratio, in fact, increased from 0.15 to 0.27. Moreover, the relative abundance of the C-O functionalities increased from 53% to 58% as a consequence of the HMF derived species adsorption. Considering our previous conclusions on the selectivity to AMF, which can be improved by confinement of the Ru particles, we supposed that the selectivity even without conversion can be improved by removing any deposit on the catalyst surface. 

**Figure 9:**
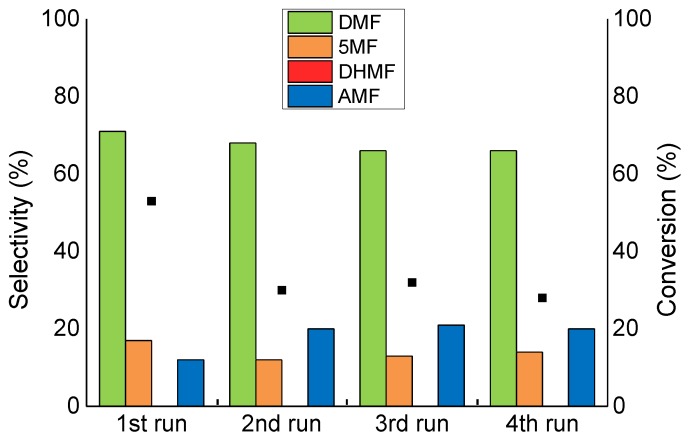
The reusability test on the Ru/AC catalyst with reactivation step. Reaction conditions: HMF, 0.08 M; substrate/metal = 100 mol/mol; total volume, 15 mL; 150 °C, 20 bar H_2_, reaction time, 1 h.

The reusability test was then repeated and a reactivation step was added (treatment at 200 °C and 5 bar of H_2_ for 1 h) in between every reaction in order to remove any adsorbed species. A small improvement was obtained (Figure 9). Although the reaction conversion dropped again from 53% to 30% after the first cycle, the catalyst activity and selectivity remained stable for further reactions. After reactivation, the XPS revealed that the adsorbed species were partially removed: the O/C ratio decreased from 0.27 to 0.21 and the relative abundance of C-O functionalities decreased back from 58% to 53% to 54% (Figure S8).

However, the sudden drop in activity after the first use cannot, therefore, be ascribed only to the adsorbed species that block the active sites. The TEM analysis on the used catalyst did not show any significant increase in the particle size (1.9 nm ± 0.5 nm, Supplementary Information) that can be attributed to the decrease in activity. The EDX analysis, however, showed a much lower amount of Ru compared to the starting nominal 1 wt% value, suggesting that the main cause of deactivation was chemical leaching.

**Figure 10:**
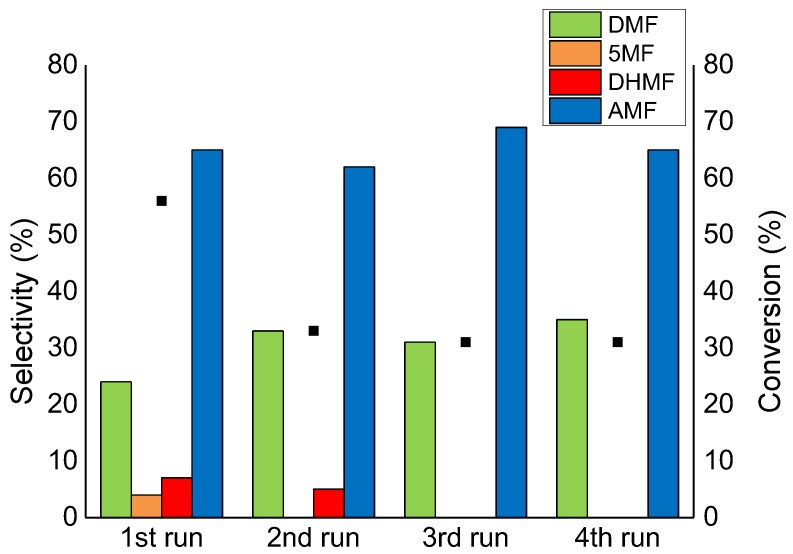
The reusability test on the Ru/CNFs-HHT catalyst. Reaction conditions: HMF, 0.08 M; substrate/metal = 100 mol/mol; total volume, 15 mL; 150 °C, 20 bar H_2_, reaction time, 1 h.

Ru/CNFs-PS and Ru/CNFs-HHT were also tested in consecutive runs. Similarly to Ru/AC, Ru/CNFs-HHT showed a loss in activity after the first use due to chemical leaching and then remained stable at ca. 30% of conversion (Figure 10). The selectivity to AMF remained stable across the four runs, while the production of DMF slightly increased; interestingly, no 5MF and DHMF were detected after the second run. The Ru/CNFs-PS catalyst, on the other hand, showed no reusability at all. The activity dropped to 0% immediately after the first run, indicating the extremely weak interaction between the metal particles and the support.

## 3. Materials and Methods 

### 3.1. Catalyst Preparation

All the catalysts were prepared by the incipient wetness impregnation method using an aqueous solution of RuCl_3_·xH_2_O (Sigma-Aldrich, Haverhill, MA, USA; 99.98%). The Norit GSX activated carbon (Alfa Aesar, Saint Louis, MO, USA; 930 m^2^ g^−1^, pore volume 0.26 mL g^−1^) and two types of pyrolytically stripped carbon fibers (PR-24-PS and PR-24-HHT, Applied Science) were used as supports. An appropriate amount of Ru precursor was dissolved in a very precise quantity of deionized water corresponding to the specific support pore volume; the pore volume of each support was obtained experimentally; these are reported in Table 3. The solution was then added dropwise into a glass vial containing the desired amount of support and stirred manually with a glass rod for a few minutes, until the formation of a very dark brown slurry. The catalysts were then washed thoroughly with 1 L of deionized water, dried at 80 °C overnight and activated before the reaction in the autoclave. The activation step consisted of a treatment at 200 °C and 5 bar of H_2_ for 1 h.

### 3.2. Hydrogenation Reactions

The hydrogenation reactions were carried out in a 100 ml stainless steel autoclave equipped with a thermocouple, a mechanical stirrer, and a glass inlet to contain the reaction solution. In a typical experiment, 15 mL of 2-butanol (Sigma-Aldrich, Haverhill, MA, USA; >99%), 0.1550 g of HMF (Sigma-Aldrich, >99%) and an appropriate amount of activated catalyst (usually 1 mol% relative to HMF) were added into a 100 ml glass inlet and placed into the autoclave. The system was flushed several times with N_2_ first and then with H_2_ and pressurized before heating. The reactor was heated at the desired temperature, setting the stirring rate to 1000 rpm, and held for a specified amount of time before the reaction was quenched in an ice bath. The catalyst was then removed by centrifugation and the filtrate was diluted with a solution of an external standard (p-xylene, Sigma-Aldrich, >99%) for GC measurement. For the analysis of the products, a GC-MS (Thermo Scientific, Waltham, MA, USA; ISQ QD equipped with an Agilent VF-5ms column) was employed and the resulting fragmentation peaks were compared with the standards present in the software database, while, for the quantification of the amounts of reactants consumed and products generated, a GC-FID equipped with a non-polar column was employed (Agilent, Santa Clara, CA, USA; 7820A equipped with an Agilent CP-Sil 5 CB column).

### 3.3. Catalysts Characterization

The TPR analysis was performed on a Thermo Scientific TPDRO 1100. The samples were firstly pretreated at 300 °C in a N_2_ flow and then analyzed in a 10% H_2_ in Ar flow using a 10 °C/min ramp up to 800 °C.

For the determination of the surface composition and oxidation state of ruthenium, X-ray photoelectron spectroscopy (XPS) measurements were done using a KRATOS XSAM 800 XPS machine. The Al K_a_ characteristic X-ray line, 40 eV pass energy, and FAT mode were applied for recording the XPS lines of the O 1s, Ru 3p, C 1s, Cl 2p regions. The C 1s binding energy at 284.5 eV was used as a reference for charge compensation. The samples were stored under air before the measurements.

High-resolution transmission electron microscopy (TEM) and high angular annular dark field scanning electron microscopy (HAADF-STEM) analyses were performed by a ZEISS LIBRA200FE microscope equipped with a FEG source operating at 200 kV, with an in-column second-generation omega filter and energy-dispersive X-ray spectroscopy (EDS – Oxford INCA Energy TEM 200). Scanning transmission electron microscopy (STEM) data were collected on the Ru catalysts by using a Hitachi H3300 STEM operated at 200 kV in the Z-contrast mode, in which the brightness depended on the thickness and the approximate square of the atomic number. The samples were dispersed by sonication in isopropanol and a drop of the suspension was deposited on a lacey-carbon film supported on a copper grid with a mesh of 300. The histograms of the metal particle size distributions were obtained by counting at least 500 particles onto the TEM micrographs. The mean particle diameter (d_m_) was calculated by using the formula d_m_ = ∑d_i_n_i_/∑n_i_, where n_i_ is the number of particles with diameter d_i_.

## 4. Conclusions

In summary, the HMF hydrogenation reaction was studied with Ru catalysts supported on carbonaceous materials. A preliminary study was conducted on a Ru/AC catalyst in order to optimise the reaction parameters such as temperature and hydrogen pressure. It was observed that the etherification mechanism was favoured at low reaction temperatures and hydrogen pressures (100 °C and 5 bar), while the hydrogenation/hydrogenolysis process dominated at higher temperatures and pressures (200 °C and 40 bar). The selectivity towards DMF or AMF could be easily switched by simply using a different carbonaceous support; DMF was, in fact, the main product with Ru/AC (75%), while AMF were predominant produced when high-temperature treated CNFs were used as supports for the Ru nanoparticles (65%). Although chemical leaching strongly limited the reusability of these catalysts, the use of carbon of a different nature as the support for Ru nanoparticles proved to be of catalytic interest in the hydrogenation of HMF for the selective production of fuels and chemicals.

## Figures and Tables

**Table 1:** The average particle size and size distribution calculated by TEM.

Catalyst	Particle Size (nm)
Ru/AC	1.7 ± 0.3
Ru/CNFs-HHT	1.0 ± 0.2
Ru/CNFs-PS	1.9 ± 1.8

**Table 2:** The XPS analysis of Ru nanoparticles supported on different carbonaceous materials. The numbers in brackets refer to the total amount of oxygen functionalities.

Catalyst		C1s					Ru 3p^3/2^	Surface Ratio	
		C-C	C-O	C=O	O-C=O	Aromatic Ring		Ru/C	O/C
Ru/AC	BE (eV)	284.5	285.8	287.2	288.7	290.3	464.8 461.2	0.009	0.15
	Rel. am. %	57	20 (53)	12 (32)	6 (16)	5	77 23		
Ru/CNFs-PS	BE (eV)	284.5	285.9	287.4	288.8	291.1	463.8	0.003	0.26
	Rel. am. %	22	32 (44)	28 (39)	12 (17)	6	-		
Ru/CNFs-HHT	BE (eV)	284.5	286.1	287.4	288.8	290.8	463.6	0.004	0.18
	Rel. am. %	37	34 (58)	18 (31)	7 (12)	5	-		
Ru/AC spent	BE (eV)	284.5	286.0	287.6	289.2	292.2	462.4	0.006	0.27
	Rel. am. %	49	29 (58)	14 (28)	7 (14)	1	-		
Ru/AC reactivated	BE (eV)	284.6	285.8	287.5	289.1	291.3	462.8	0.006	0.21
	Rel. am. %	31	35 (54)	21 (32)	9 (14)	3	-		

**Table 3:** The specific support pore volume.

Support	Pore Volume (mL/g)
AC	0.26
CNFs-PS	1.45
CNFs-HHT	1.50

## Supplementary Materials

The Figures S1–S6 are available online.

## References

[1] (2014). BP Statistical Review of World Energy June 2014.

[2] Binder J.B., Raines R.T., Binder J.B., Raines R.T. (2009). Simple Chemical Transformation of Lignocellulosic Biomass into Furans for Fuels and Chemicals. J. Am. Chem. Soc..

[3] Cao Q., Guo X., Guan J., Mu X., Zhang D. (2011). A process for efficient conversion of fructose into 5-hydroxymethylfurfural in ammonium salts. Appl. Catal. A Gen..

[4] Perego C., Bosetti A. (2011). Biomass to fuels: The role of zeolite and mesoporous materials. Microporous Mesoporous Mater..

[5] Qi X., Guo H., Li L. (2011). Efficient conversion of fructose to 5-hydroxymethylfurfural catalyzed by sulfated zirconia in ionic liquids. Ind. Eng. Chem. Res..

[6] Wang P., Yu H., Zhan S., Wang S. (2011). Catalytic hydrolysis of lignocellulosic biomass into 5-hydroxymethylfurfural in ionic liquid. Bioresour. Technol..

[7] Serrano-Ruiz J.C., Dumesic J.A. (2012). Catalytic production of liquid hydrocarbon transportation fuels. Catal. Altern. Energy Gener..

[8] Qi X., Watanabe M., Aida T.M., Smith R.L. (2012). Synergistic conversion of glucose into 5-hydroxymethylfurfural in ionic liquid-water mixtures. Bioresour. Technol..

[9] Van Putten R.J., Van Der Waal J.C., De Jong E., Rasrendra C.B., Heeres H.J., De Vries J.G. (2013). Hydroxymethylfurfural, a versatile platform chemical made from renewable resources. Chem. Rev..

[10] Kroh L.W. (1994). Caramelisation in food and beverages. Food Chem..

[11] Rosatella A.A., Simeonov S.P., Frade R.F.M., Afonso C.A.M. (2011). 5-Hydroxymethylfurfural (HMF) as a building block platform: Biological properties, synthesis and synthetic applications. Green Chem..

[12] Wu S., Fan H., Xie Y., Cheng Y., Wang Q., Zhang Z., Han B. (2010). Effect of CO_2_ on conversion of inulin to 5-hydroxymethylfurfural and propylene oxide to 1{,}2-propanediol in water. Green Chem..

[13] Mitra J., Zhou X., Rauchfuss T. (2015). Pd/C-catalyzed reactions of HMF: Decarbonylation, hydrogenation, and hydrogenolysis. Green Chem..

[14] Luo J., Arroyo-Ramírez L., Wei J., Yun H., Murray C.B., Gorte R.J. (2015). Comparison of HMF hydrodeoxygenation over different metal catalysts in a continuous flow reactor. Appl. Catal. A Gen..

[15] Hales R.A. (1962). Process for Preparing 2,5-bis Hydroxymethyl Tetrahydrofuran. U.S. Patent.

[16] Tuteja J., Choudhary H., Nishimura S., Ebitani K. (2014). Direct synthesis of 1,6-hexanediol from HMF over a heterogeneous Pd/ZrP catalyst using formic acid as hydrogen source. ChemSusChem.

[17] Yao S., Wang X., Jiang Y., Wu F., Chen X., Mu X. (2014). One-step conversion of biomass-derived 5-hydroxymethylfurfural to 1,2,6-hexanetriol over ni–co–al mixed oxide catalysts under mild conditions. ACS Sustain. Chem. Eng..

[18] Luo J., Yu J., Gorte R.J., Mahmoud E., Vlachos D.G., Smith M.A. (2014). The effect on oxide acidity on HMF etherification. Catal. Sci. Technol..

[19] Wang C., Xu H., Daniel R., Ghafourian A., Herreros J.M., Shuai S., Ma X. (2013). Combustion characteristics and emissions of 2-methylfuran compared to 2,5-dimethylfuran, gasoline and ethanol in a DISI engine. Fuel.

[20] Román-Leshkov Y., Barrett C.J., Liu Z.Y., Dumesic J.A. (2007). Production of dimethylfuran for liquid fuels from biomass-derived carbohydrates. Nature.

[21] Nagpure A.S., Venugopal A.K., Lucas N., Manikandan M., Thirumalaiswamy R., Chilukuri S. (2015). Renewable fuels from biomass-derived compounds: Ru-containing hydrotalcites as catalysts for conversion of HMF to 2,5-dimethylfuran. Catal. Sci. Technol..

[22] Zu Y., Yang P., Wang J., Liu X., Ren J., Lu G., Wang Y. (2014). Efficient production of the liquid fuel 2,5-dimethylfuran from 5-hydroxymethylfurfural over Ru/Co_3_O_4_ catalyst. Appl. Catal. B Environ..

[23] Jae J., Zheng W., Lobo R.F., Vlachos D.G. (2013). Production of dimethylfuran from hydroxymethylfurfural through catalytic transfer hydrogenation with ruthenium supported on carbon. ChemSusChem.

[24] Jae J., Mahmoud E., Lobo R.F., Vlachos D.G. (2014). Cascade of liquid-phase catalytic transfer hydrogenation and etherification of 5-hydroxymethylfurfural to potential biodiesel components over Lewis acid zeolites. ChemCatChem.

[25] Hu L., Tang X., Xu J., Wu Z., Lin L., Liu S. (2014). Selective transformation of 5-hydroxymethylfurfural into the liquid fuel 2,5-dimethylfuran over carbon-supported ruthenium. Ind. Eng. Chem. Res..

[26] Thananatthanachon T., Rauchfuss T.B. (2010). Efficient production of the liquid fuel 2,5-dimethylfuran from fructose using formic acid as a reagent. Angew. Chem..

[27] Shi J., Wang Y., Yu X., Du W., Hou Z. (2016). Production of 2,5-dimethylfuran from 5-hydroxymethylfurfural over reduced graphene oxides supported Pt catalyst under mild conditions. Fuel.

[28] Kong X., Zheng R., Zhu Y., Ding G., Zhu Y., Li Y.-W. (2015). Rational design of Ni-based catalysts derived from hydrotalcite for selective hydrogenation. Green Chem..

[29] Zhu Y., Kong X., Zheng H., Ding G., Zhu Y., Li Y.-W. (2015). Efficient synthesis of 2,5-dihydroxymethylfuran and 2,5-dimethylfuran from 5-hydroxymethylfurfural using mineral-derived Cu catalysts as versatile catalysts. Catal. Sci. Technol..

[30] Nishimura S., Ikeda N., Ebitani K. (2014). Selective hydrogenation of biomass-derived 5-hydroxymethylfurfural (HMF) to 2,5-dimethylfuran (DMF) under atmospheric hydrogen pressure over carbon supported PdAu bimetallic catalyst. Catal. Today.

[31] Wang G.-H., Hilgert J., Richter F.H., Wang F., Bongard H.-J., Spliethoff B., Weidenthaler C., Schüth F. (2014). Platinum-cobalt bimetallic nanoparticles in hollow carbon nanospheres for hydrogenolysis of 5-hydroxymethylfurfural. Nat. Mater..

[32] Luo J., Yun H., Mironenko A.V., Goulas K., Lee J.D., Monai M., Wang C., Vorotnikov V., Murray C.B., Vlachos D.G. (2016). Mechanisms for High Selectivity in the Hydrodeoxygenation of 5-Hydroxymethylfurfural over PtCo Nanocrystals. ACS Catal..

[33] Luo J., Monai M., Wang C., Lee J.D., Duchoň T., Dvořák F., Matolín V., Murray C.B., Fornasiero P., Gorte R.J. (2017). Unraveling the surface state and composition of highly selective nanocrystalline Ni-Cu alloy catalysts for hydrodeoxygenation of HMF. Catal. Sci. Technol..

[34] Bicker M., Kaiser D., Ott L., Vogel H. (2005). Dehydration of d-fructose to hydroxymethylfurfural in sub- and supercritical fluids. J. Supercrit. Fluids.

[35] Gruter G.J.M., Manzer L.E., De Sousa Dias A.S.V., Dautzenberg F., Purmova J. (2012). Hydroxymethylfurfural Ethers and Esters Prepared in Ionic Liquids. U.S. Patent.

[36] Gruter G.J.M., Dautzenberg F. (2012). Method for the Synthesis of S-Alkoxymethyl Furfural Ethers and Their Use. U.S. Patent.

[37] Mascal M., Nikitin E.B. (2008). Direct, high-yield conversion of cellulose into biofuel. Angew. Chem. Int. Ed. Engl..

[38] Balakrishnan M., Sacia E.R., Bell A.T. (2012). Etherification and reductive etherification of 5-(hydroxymethyl)furfural: 5-(alkoxymethyl)furfurals and 2{,}5-bis(alkoxymethyl)furans as potential bio-diesel candidates. Green Chem..

[39] Lanzafame P., Temi D.M., Perathoner S., Centi G., Macario A., Aloise A., Giordano G. (2011). Etherification of 5-hydroxymethyl-2-furfural (HMF) with ethanol to biodiesel components using mesoporous solid acidic catalysts. Catal. Today.

[40] Cao Q., Liang W., Guan J., Wang L., Qu Q., Zhang X., Wang X., Mu X. (2014). Catalytic synthesis of 2,5-bis-methoxymethylfuran: A promising cetane number improver for diesel. Appl. Catal. A Gen..

[41] Alipour S., Omidvarborna H., Kim D.S. (2017). A review on synthesis of alkoxymethyl furfural, a biofuel candidate. Renew. Sustain. Energy Rev..

[42] Chheda J.N., Huber G.W., Dumesic J.A. (2007). Liquid-phase catalytic processing of biomass-derived oxygenated hydrocarbons to fuels and chemicals. Angew. Chem. Int. Ed..

[43] Li J., Liu J.L., Zhou H.J., Fu Y. (2016). Catalytic Transfer Hydrogenation of Furfural to Furfuryl Alcohol over Nitrogen-Doped Carbon-Supported Iron Catalysts. ChemSusChem.

[44] Rossetti I., Pernicone N., Forni L. (2003). Characterisation of Ru/C catalysts for ammonia synthesis by oxygen chemisorption. Appl. Catal. A Gen..

[45] Peng G., Steib M., Ludwig C., Gramm F., Vogel F. (2014). Synthesis factors affecting the catalytic performance and stability of Ru/C catalysts for supercritical water gasification. Catal. Sci. Technol..

[46] Guerrero-Ruiz A., Badenes P., Rodríguez-Ramos I. (1998). Study of some factors affecting the Ru and Pt dispersions over high surface area graphite-supported catalysts. Appl. Catal. A Gen..

[47] Villa A., Schiavoni M., Chan-Thaw C.E., Fulvio P.F., Mayes R.T., Dai S., More K.L., Veith G.M., Prati L. (2015). Acid-Functionalized Mesoporous Carbon: An Efficient Support for Ruthenium-Catalyzed γ-Valerolactone Production. ChemSusChem.

[48] Prati L., Bergna D., Villa A., Spontoni P., Bianchi C.L., Hu T., Romar H., Lassi U. (2018). Carbons from second generation biomass as sustainable supports for catalytic systems. Catal. Today.

[49] Yu L., He L., Chen J., Zheng J., Ye L., Lin H., Yuan Y. (2015). Robust and recyclable nonprecious bimetallic nanoparticles on carbon nanotubes for the hydrogenation and hydrogenolysis of 5-hydroxymethylfurfural. ChemCatChem.

