# Supplementary material for: Ruthenium on Carbonaceous Materials for the Selective Hydrogenation of HMF
Source: Molecules. 2018 Aug 11;23(8):2007. doi: 10.3390/molecules23082007 (PMC6222502; doi:10.3390/molecules23082007)

Supplementary Information

Ruthenium on carbonaceous materials for the selective hydrogenation of HMF

Stefano Cattaneo [1], Hadi Naslhajian [2], Ferenc Somodi [3], Claudio Evangelisti [4], Alberto Villa [1] and Laura Prati [1,*]

[1] Dipartimento di Chimica, Università degli Studi di Milano, via Golgi 19, I-20133 Milano, Italy;
[2] School of Chemistry, Collage of Science, University of Tehran, P.O. Box 14155-6619, Tehran, Iran;
[3] Centre for Energy Research, Hungarian Academy of Science, Department of Surface Chemistry and Catalysis, Konkoly-Thege M. street 29-33, 1121 Budapest, Hungary;
[4] National Council of the Research, CNR-ISTM, Via G. Fantoli 16/15, 20138 Milan, Italy;
* Correspondence: laura.prati@unimi.it; Tel.: ++39–0250-314-357

Figure S1. (a) STEM micrograph of the Ru/CNFs-PS catalyst with relative (b) particle size distribution. (c) HRTEM micrograph of the Ru/CNFs-HHT catalyst with relative (d) particle size distribution. (e) Particle size distribution of the Ru/AC catalyst.

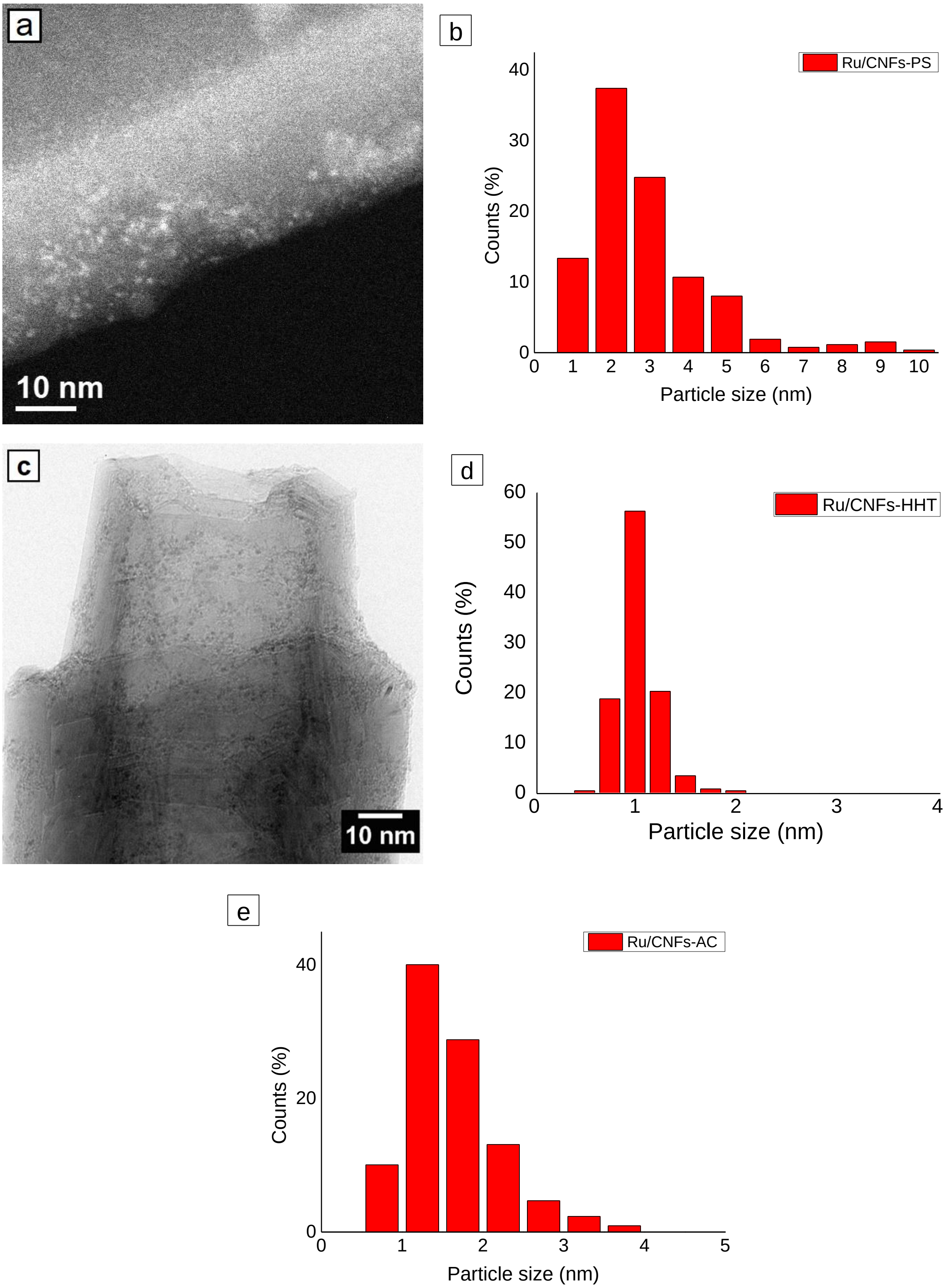

Figure S2. XPS analysis of the three fresh catalysts used in the HMF hydrogenation reaction. (a) Ru 3p$^{3/2}$, (b) O 1s and (c) C 1S.

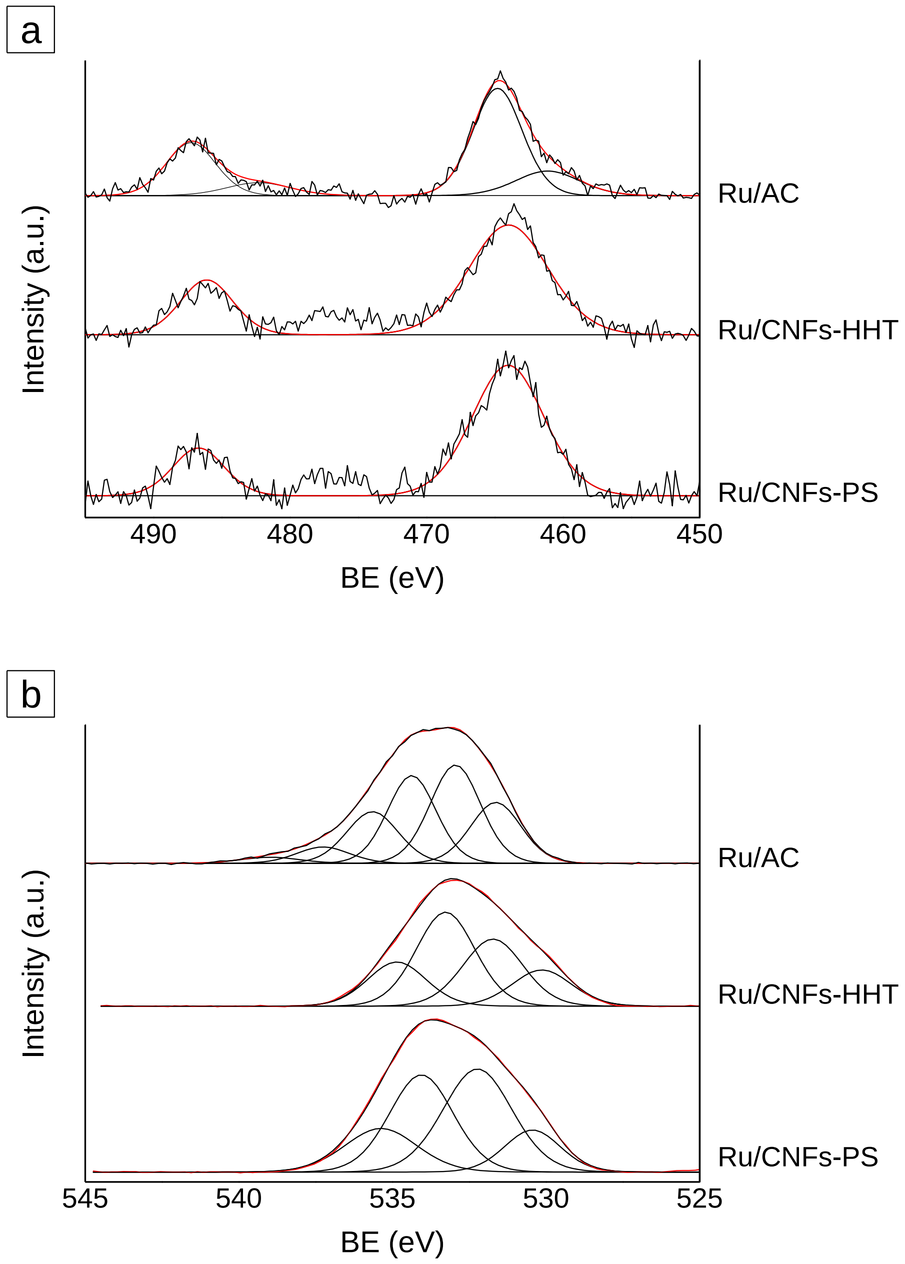

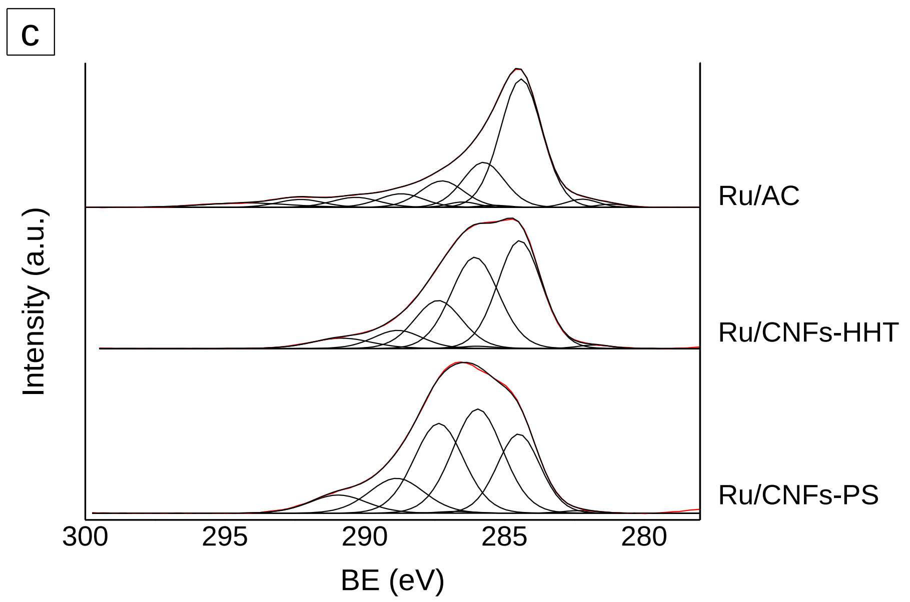
c
Intensity (a.u.)
Ru/AC
Ru/CNFs-HHT
Ru/CNFs-PS
300
295
290
285
280
BE (eV)

Figure S3. Products of etherification of dihydroxymethylfuran and furfuryl alcohol with the solvent 2-butanol.

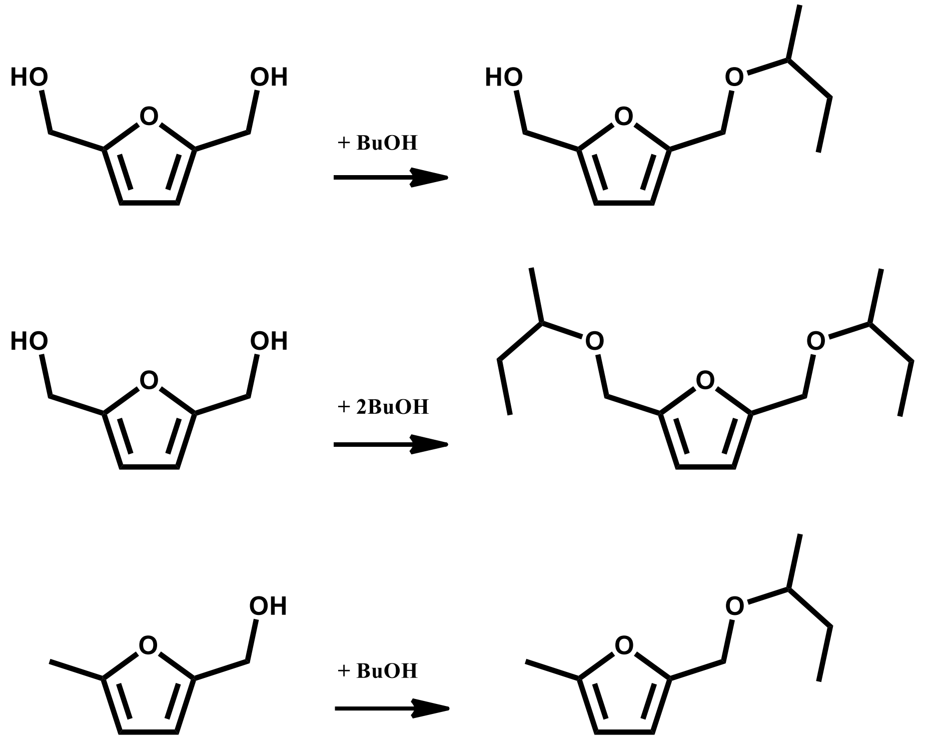

Figure S4. GC-MS analysis of the AMF detected in the reaction mixture.

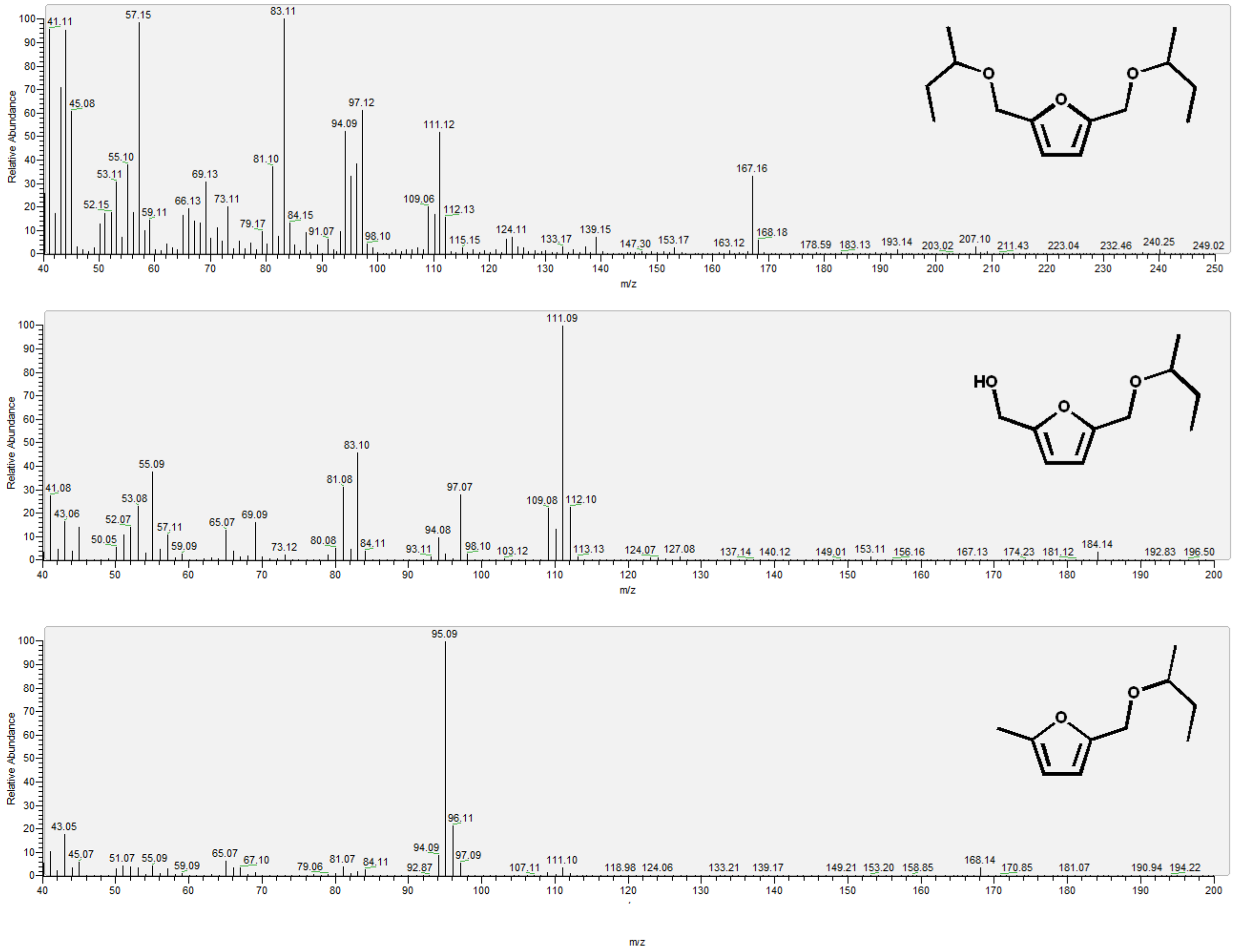

Figure S5. XPS analysis of the Ru/AC before reaction, after reaction and after reactivation. (a) Ru $3p^{3/2}$, (b) O 1s and (c) C 1S.

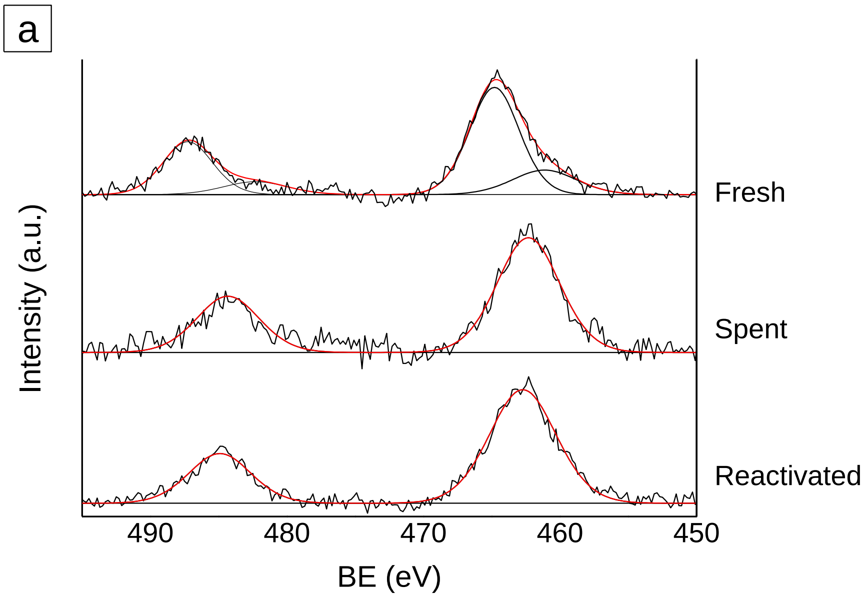

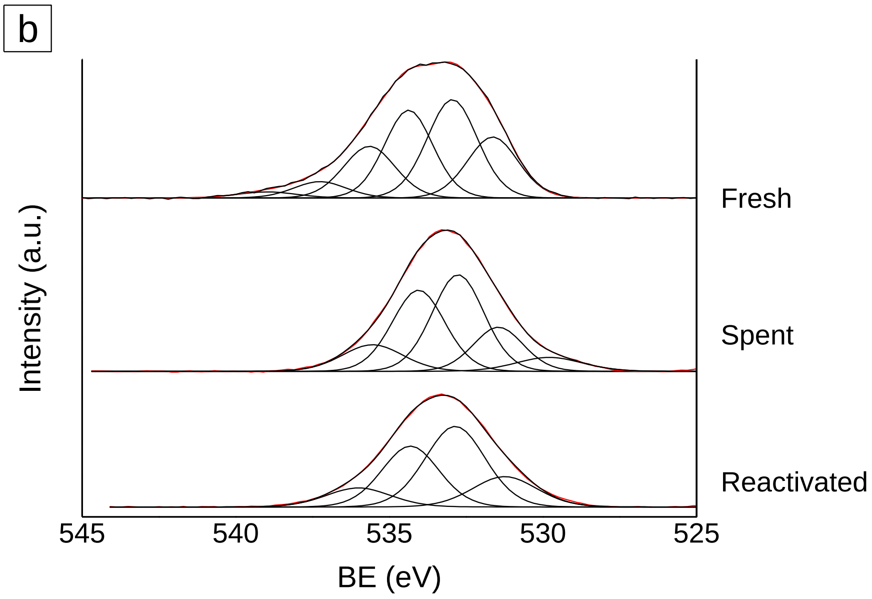

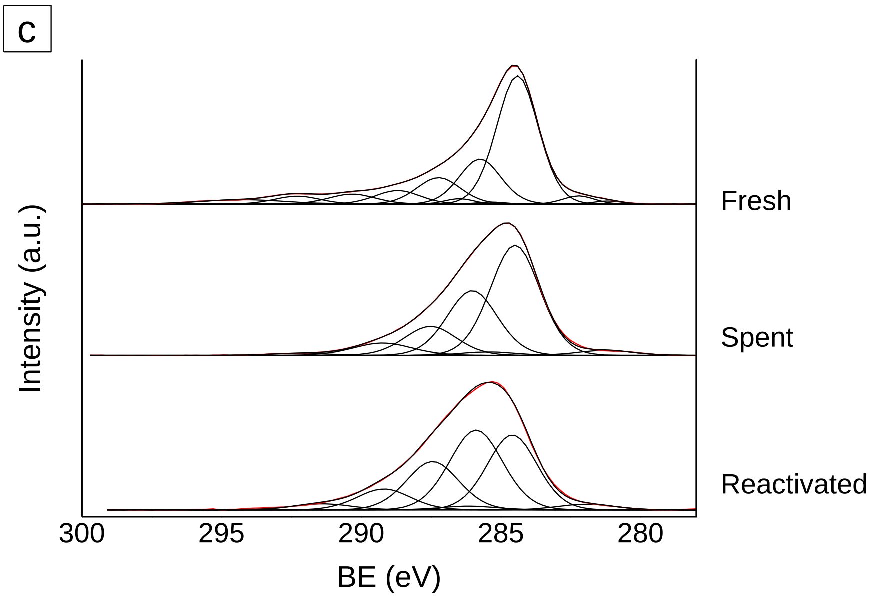
c
Intensity (a.u.)
Fresh
Spent
Reactivated
300
295
290
285
280
BE (eV)

Figure S6. HRTEM micrograph of the spent Ru/AC catalyst with relative particle size distribution.

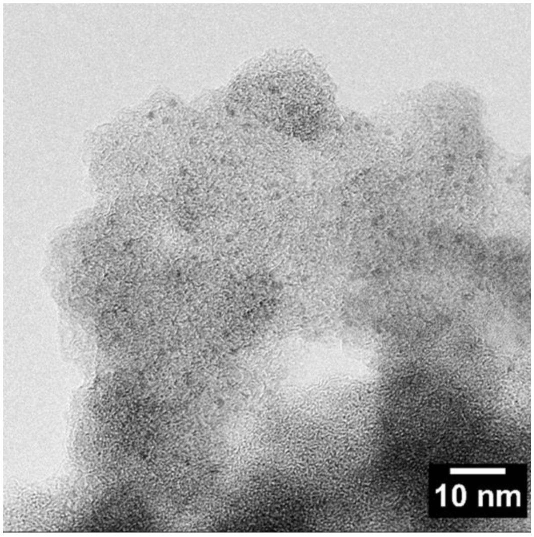

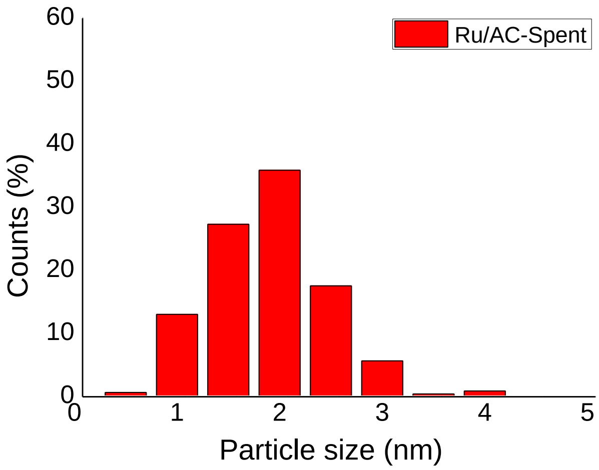

Supplement: Supplementary file 1 [file molecules-23-02007-s001.pdf]
